# WNT and NOTCH signaling pathways as activators for epidermal growth factor receptor in esophageal squamous cell carcinoma

**DOI:** 10.1186/s11658-018-0109-x

**Published:** 2018-09-03

**Authors:** Mohammad Reza Abbaszadegan, Anali Riahi, Mohammad Mahdi Forghanifard, Meysam Moghbeli

**Affiliations:** 1 Medical Genetics Research Center, Faculty of Medical Sciences, Mashhad University of Medical Sciences, Mashhad, Iran; 2 Immunology Research Center, Mashhad University of Medical Sciences, Mashhad, Iran; 3 Department of Biology, Damghan Branch, Islamic Azad University, Damghan, Iran; 4 Department of Modern Sciences and Technologies, Faculty of Medicine, Mashhad University of Medical Sciences, Mashhad, Iran

**Keywords:** Growth factor, WNT, NOTCH, Early stage, Survival

## Abstract

**Background:**

Esophageal squamous cell carcinoma (ESCC) is the most common histological type of esophageal cancer, with a poor prognosis. Deregulation of WNT and NOTCH signaling pathways is important in ESCC progression, which can be due to either malfunction of their components or crosstalk with other pathways. Therefore, identification of new crosstalk between such pathways may be effective to introduce new strategies for targeted therapy of cancer. A correlation study was performed to assess the probable interaction between growth factor receptors and WNT/NOTCH pathways via the epidermal growth factor receptor (EGFR) and Musashi1 (MSI1), respectively.

**Methods:**

Levels of MSI1/EGFR mRNA expression in tumor tissues from 48 ESCC patients were compared to their corresponding normal tissues using real-time polymerase chain reaction.

**Results:**

There was a significant correlation between EGFR and MSI1 expression (*p* = 0.05). Moreover, there was a significant correlation between EGFR/MSI1 expression and grade of tumor differentiation (*p* = 0.02).

**Conclusion:**

This study confirms a direct correlation between MSI1 and EGFR and may support the important role of MSI1 in activation of EGFR through NOTCH/WNT pathways in ESCC.

## Background

Esophageal cancer is the sixth leading cause of cancer-related deaths worldwide [1]. Esophageal squamous cell carcinoma (ESCC) is the most common histological type of esophageal carcinoma globally [2]. It represents more than 95% of esophageal cancers in Asia [3]. Although ESCC is the most common histological type in low-resource areas, adenocarcinoma represents 20–50% of the cases in Western countries [4]. In the case of ESCC incidence, there are geographical variations with a hot spot in the “Asian esophageal cancer belt” spreading from the Caspian Sea to central China [5]. Since ESCC is diagnosed at advanced stages of the disease, it usually has a poor prognosis. Despite the recent progress in chemoradiotherapeutic modalities, ESCC still has a five-year survival rate less than 20% [6–8]. Epidermal growth factor receptor (EGFR) is a transmembrane protein composed of an extracellular ligand-binding and an intracellular tyrosine kinase domain. Following ligand binding, EGFR experiences a conformational change and induces dimerization with other ErbB/HER family receptors leading to autophosphorylation and activation of the tyrosine kinase domain [9]. Subsequently, several pathways such as ERK/MAPK, PI3K, and JAK/STAT can be activated to regulate cell proliferation and migration [10, 11]. An inverse association has been shown between EGFR expression and survival rate of ESCC patients in which higher levels of EGFR are associated with chemo-radiotherapeutic resistance and lymph node metastasis. EGFR overexpression is involved in higher cell proliferation and metastasis [12, 13]. WNT signaling is one of the most important pathways in both embryogenesis and tumorigenesis [14]. WNT signals stabilize cytoplasmic beta-catenin via the Frizzled/LRP5/6 complex through preventing its phosphorylation-dependent degradation. It has been shown that EGFR is a direct target of the WNT pathway, and EGFR activation is associated with some proliferative effects of increased beta-catenin [15]. EGFR activates beta-catenin through PI3K/Akt in tumor cells [16, 17]. Musashi1 (Msi1) is an RNA-binding protein (RBP) with two tandem RNA recognition motifs located in the 3′ untranslated region (UTR). MSI1 exerts its inhibitory role through competing with eIF4G to bind PABP during initiation of translation [18]. It targets different RNAs such as Numb and p21WAF-1, which are involved in the NOTCH pathway and cell cycle regulation, respectively [19]. Numb is a suppressor for several pathways such as Hedgehog and NOTCH [20, 21]. Also DKK3, as one of the main targets for the post-transcriptional regulation of MSI1, functions as a tumor suppressor to block proliferation through interaction with LRP5/6 [14, 22]. It prevents beta-catenin transfer into the nucleus [23]. Moreover, the WNT pathway regulates NUMB through a TCF/LEF binding site within the NUMB promoter [24]. Therefore, MSI1 overexpression may be associated with the Notch-1 and WNT signaling pathways. In the present study we assessed a probable mutual correlation between EGFR and MSI1 to clarify the details of interactions between WNT and NOTCH pathways and their probable effect on EGFR in ESCC patients.

## Methods

### Tissue samples

Forty-eight new case ESCC patients who had not received any chemo-radiotherapeutic modalities before surgery were enrolled in this study. Tumor tissues were examined histologically and were found to comprise at least 70% tumor cells. These cases were gathered from the Qaem and Imam Reza hospitals of Mashhad University of Medical Sciences (2010–2015). Informed consent forms were also signed by the patients.

### RNA extraction, cDNA synthesis, and quantitative RT-PCR

Total RNA was extracted from the normal and tumor fresh tissues using the RNeasy Mini Kit (Qiagen, Germany). Subsequently, cDNA synthesis performed using the First-Strand Synthesis kit (Fermentas, Lithuania). Finally, the levels of EGFR and MSI1 mRNA expression in normal and fresh tissues were assessed using comparative relative real-time PCR (SYBR Green, AMPLIQON, Denmark; Stratagene Mx-3000P, USA) in duplicate reactions with specific primer sequences [25, 26]). Glyceraldehyde 3-phosphate dehydrogenase (GAPDH) was used as a normalizer [27].

### Statistical analysis

Spearman’s q and Pearson v-squared were used to evaluate the probable correlation between EGFR and MSI1 expression. ANOVA and t-test were also used to assess the correlations between EGFR/MSI1 expression and clinicopathological features of patients (*p* < 0.05). All the statistical analysis was performed using SPSS 16.0 (SPSS, Chicago, IL).

## Results

### Study population

Forty-eight ESCC patients comprising 28 (58.3%) males and 20 (41.7%) females were enrolled in this study. The age range was 30–83 years with mean age ± SD of 61.85 ± 1.78 years and tumor size ranged from 1.5 to 12 cm with mean size ±SD of 4.23 ± 0.28 cm. Most of the tumors were located in the middle esophagus (27; 56.2%), moderately differentiated (31; 64.6%), had tumor stage of II (28; 58.3%), and T3 depth of invasion (40; 83.3%) (Table 1).

**Table 1:** EGFR/MSI1 mRNA expression and clinicopathological features of ESCC patients

	Total	EGFR/ MSI1 over expression	EGFR/ MSI1 under expression	EGFR/ MSI1 normal expression	EGFR over expression	MSI1 over expression	*P- Value*
Patients	48	7(14.6%)	2(4.2%)	14(29.2%)	12(25%)	12(25%)	
Mean age (mean ± SD)	61.85 ± 12.33	55.86 ± 5.65	42.50 ± 6.50	66.43 ± 2.30	63.17 ± 2.36	60.58 ± 4.29	
Size (mean ± SD)	4.23 ± 1.91	3.50 ± 0.58	3.75 ± 1.75	4.20 ± 0.36	4.29 ± 0.58	4.88 ± 0.73	
Sex							0.553
Male	28(58.3%)	5(71.4%)	–	8(57.1%)	8(66.7%)	7(58.3%)	
Female	20(41.7%)	2(28.6%)	2(100%)	6(42.9%)	4(33.3%)	5(41.7%)	
Location							0.226
Lower	21(43.8%)	2(28.6%)	–	8(57.1%)	4(33.3%)	6(50%)	
Middle	27(56.2%)	5(71.4%)	2(100%)	6(42.9%)	8(66.7%)	6(50%)	
Grade							**0.020**
Poorly differentiated	8(16.7%)	2(28.6%)	2(100%)	1(7.1%)	1(8.3%)	1(8.3%)	
Moderately differentiated	31(64.6%)	5(71.4%)	–	11(78.6%)	8(66.7%)	7(58.3%)	
Well differentiated	9(18.8%)	–	–	2(14.3%)	3(25%)	4(33.3%)	
Lymph node metastasis							0.702
Yes	22(45.8%)	2(28.6%)	1(50%)	8(57.1%)	4(33.3%)	6(50%)	
No	26(54.2%)	5(71.4%)	1(50%)	6(42.9%)	8(66.7%)	6(50%)	
Stage							0.763
I/II	29(60.4%)	5(71.4%)	1(50%)	7(50%)	9(75%)	7(58.3%)	
III/IV	19(39.6%)	2(28.6%)	1(50%)	7(50%)	3(25%)	5(41.7%)	
Depth of tumor invasion (T)							0.292
T1	1(2.1%)	1(14.3%)	–	–	–	–	
T2	7(14.6%)	–	–	2(14.3%)	4(33.3%)	1(8.3%)	
T3	40(83.3%)	6(85.7%)	2(100%)	12(85.7%)	8(66.7%)	11(91.7%)	

Bold values indicate significant correlation between mRNA expression and clinicopathological features

### Levels of EGFR/MSI1 mRNA expression in ESCC patients

**Fig. 1:**
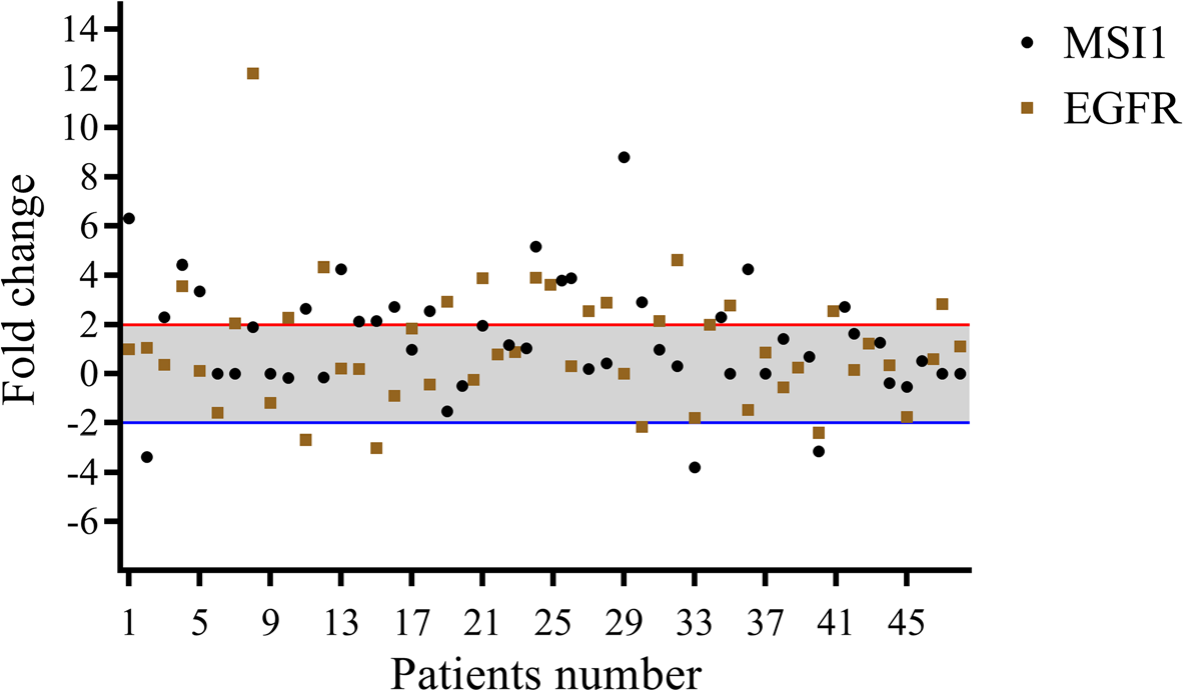
Descriptive analysis of relative gene expression of MSI1 and EGFR in ESCC patients. The thresholds for the over- and underexpressed cases are shown by red and blue lines, respectively. The grey area refers to the cases with normal levels of EGFR and MSI1 mRNA expression

Regarding our recent publications, we have reported the role of EGFR and MSI1 in separate studies [25, 26]. Here, a probable mutual correlation between such markers was assessed for the first time among ESCC patients. Seven out of 48 cases (14.6%) had overexpression of both of these markers, whereas only two cases (4.2%) showed simultaneous underexpression. Twenty-one out of 48 patients (43.7%) had tumors with overexpression in just one of these markers. Moreover, 14/48 (29.2%) cases were normal for EGFR and MSI1 expression (Table 1). There was a significant correlation between EGFR/MSI1 mRNA expression, in which mean fold of MSI1 mRNA expression in EGFR underexpressed cases was significantly higher than that in the EGFR overexpressed cases (1.47 ± 0.48 vs. 1.31 ± 0.42, fold changes) (*p* = 0.05). EGFR and MSI1 fold changes are presented in a scatter plot (Fig. 1).

### Clinicopathological features and EGFR/MSI1 mRNA expression

There was a significant correlation between EGFR/MSI1 mRNA expression and grade of tumor differentiation in which 5/7 (71.43%) tumors with concomitant overexpression of these markers were moderately differentiated (*p* = 0.02). There was no significant correlation between tumor location and EGFR/MSI1 expression, but the majority of EGFR/MSI1 overexpressed cases were located in the middle esophagus (5/7, 71.4%). Although there was no significant correlation between tumor depth of invasion and EGFR/MSI1 expression, most of the EGFR/MSI1 overexpressed cases had T3 depth of invasion (6/7, 85.7%). We did not observe any significant correlation between expression of these markers and other clinicopathological features of patients including sex, lymph node metastasis, or tumor stage; however, the majority of EGFR/MSI1 overexpressed cases did not have any metastatic lymph nodes, were in tumor stage I/II, and were observed in males (5/7, 71.4%). In the case of tumor size, the biggest and smallest tumors were observed among patients with EGFR underexpression or MSI1 overexpression and patients with EGFR/MSI1 overexpression, respectively (5.67 ± 3.18 and 3.5 ± 0.58 cm). The youngest and oldest patients had underexpression in EGFR/MSI1 and normal expression in both of these markers, respectively (42.5 ± 6.5 and 66.43 ± 2.3 years old). Although there was no significant correlation between levels of EGFR/MSI1 mRNA expression and sex, the expression of both these markers was noticeably higher in males than in females (2.08 ± 0.43 vs. 0.46 ± 0.48, MSI1 fold changes; 1.50 ± 0.53 vs. 0.52 ± 0.45, EGFR fold changes). Tumor tissues had a rising trend of MSI1 mRNA expression from a poorly to well-differentiated state, showing the importance of MSI1 in the differentiation process, but there was not a similar trend for EGFR expression and tumor grade. The highest levels of EGFR mRNA expression were observed among the moderately differentiated tumors (1.44 ± 0.50, fold changes). Levels of EGFR and MSI1 mRNA expression were higher in tumor stage I/II in comparison with stage III/IV, whereas the levels of MSI1 expression were higher than the EGFR expression in patients with tumor stage I/II (1.66 ± 0.45 vs. 1.41 ± 0.52, fold changes). These markers showed different patterns of expression based on tumor depth of invasion, in which the MSI1 expression in T3 was higher than that in T2 (1.48 ± 0.39 vs. 0.55 ± 0.49, fold changes), whereas the EGFR mRNA expression in T2 tumors was higher than that in T3 tumors (1.43 ± 0.84 vs. 0.97 ± 0.41, fold changes). In the case of lymph node metastasis, the levels of MSI1 mRNA expression were higher than the EGFR expression in tumors with metastatic lymph nodes (1.02 ± 0.46 vs. 0.61 ± 0.47, fold changes).

## Discussion

**Fig. 2:**
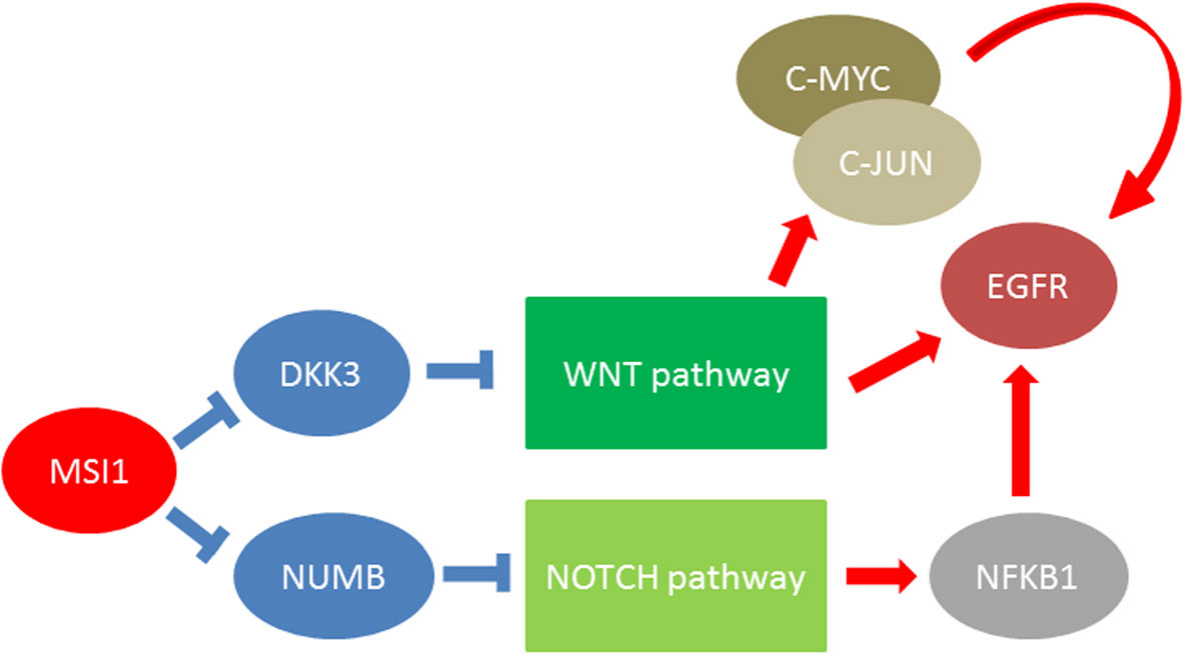
Probable correlation between MSI1 and EGFR through the WNT and NOTCH pathways

The present study was in line with our previous projects about EGFR and MSI1 in ESCC patients to find a probable correlation between these markers and introduce a panel of diagnostic markers [14, 25, 26, 28–30]. We have recently reported a direct correlation between PYGO2, the main transcription factor of the WNT pathway, and EGFR, one of the target genes in the WNT pathway [25]. On the other hand, EGFR also regulates beta-catenin stability in cells, providing positive feedback for PYGO2 [31]. Most EGFR alterations in ESCC can be observed by EGFR gene amplification and protein overexpression [32–35]. However, expression of EGFR in ESCC patients ranges between 4 and 86% [33–35]. Deregulation of EGFR plays an important role in tumor progression of lung [36], breast [37], gastrointestinal [38], and liver carcinoma [39]. Musashi1 is also expressed in different malignancies such as colorectal [40], endometrial [41], bladder [42], and esophageal cancers [43]. Regarding the present study, there was a significant correlation between these markers, in which the level of EGFR mRNA expression in tumors with MSI1 overexpression was higher than that in the normal/under expressed cases (1.16 ± 0.75 vs. 1.04 ± 0.34, fold changes). The promoter sequence of EGFR has several binding sites for NFKB1, which is one of the target genes in the NOTCH pathway; therefore activation of MSI1 may activate the NOTCH pathway via suppression of NUMB and subsequently result in activation of EGFR. Moreover, C-MYC and C-JUN, as the main target genes of the WNT pathway, also have binding sites in the promoter sequence of EGFR. Therefore, MSI1 may upregulate EGFR expression directly through the WNT pathway and indirectly through mediators such as NFKB1, C-FOS, and C-JUN, which have binding sites in the promoter sequence of EGFR and are target genes in WNT and NOTCH pathways (Fig. 2). It was shown that the expression of both of these markers is higher in the primary stages of tumors, indicating the importance of these factors in primary steps of tumor progression. Therefore, EGFR/MSI1 may be used as efficient diagnostic markers in primary stages of ESCC. In contrast, there was a reverse correlation between MSI1/EGFR expression and tumor depth of invasion, in which MSI1 is involved in T3 depth of invasion, whereas the EGFR is involved in T2 depth of invasion. With the increasing depths of ESCC tumor invasion there was a declining and rising trend in EGFR and MSI1 expression, respectively. There was a significant correlation between EGFR/MSI1 expression and tumor differentiation grade. However, we observed different patterns of mRNA expression in these markers toward the higher grades of differentiation, in which MSI1 expression had a direct and constant trend toward the well-differentiated tumors but there was no trend in EGFR expression in the case of tumor differentiation. Therefore, simultaneous overexpression leads to moderate differentiation, whereas MSI1 overexpression stimulates the tumor into differentiation toward the well-differentiated state. EGFR expression did not have any noticeable influence on the differentiation process in ESCC patients. Furthermore, there was an interesting observation concerning EGFR/MSI1 expression and sex showing that the levels of mRNA expressions in both of these markers in males were higher than in females, which needs further assessment.

## Conclusions

There are complicated interactions between the tumor markers, heralding a new era to study the biology of tumor cells in greater detail to introduce new options for targeted therapies. This correlational study of EGFR/MSI1 mRNA expression confirms a direct correlation between such markers and may support the important role of MSI1 in activation of EGFR through NOTCH/WNT pathways in ESCC. Moreover, overexpression of both markers in early stages of disease may be efficiently used for the early detection of ESCC. Indeed, early detection will be a critical step to better management of disease by selection of a proper method of treatment for ESCC patients.
